# Circadian Rhythms, Sleep, Immunity, and Fragility in the Elderly: The Model of the Susceptibility to Infections

**DOI:** 10.3389/fneur.2020.558417

**Published:** 2020-12-18

**Authors:** Sergio Garbarino, Paola Lanteri, Walter G. Sannita, Nicola L. Bragazzi, Egeria Scoditti

**Affiliations:** ^1^Department of Neuroscience, Rehabilitation, Ophthalmology, Genetics and Maternal/Child Sciences, Polyclinic Hospital San Martino Istituto di Ricovero e Cura a Carattere Scientifico (IRCCS), University of Genova, Genova, Italy; ^2^Department of Diagnostics and Applied Technology, Neurophysiopathology Center, Fondazione Istituto di Ricovero e Cura a Carattere Scientifico (IRCCS), Istituto Neurologico Carlo Besta, Milan, Italy; ^3^Laboratory for Industrial and Applied Mathematics, Department of Mathematics and Statistics, York University, Toronto, ON, Canada; ^4^National Research Council, Institute of Clinical Physiology, Lecce, Italy

**Keywords:** aging, circadian rhythm, clock gene, chronotherapy, healthcare, infection, immunity, sleep

## Immunosenescence and Elderly Frailty

The ongoing pandemic of COVID-19, caused by the severe acute respiratory syndrome coronavirus (SARS-CoV-2), has dramatically shown how the elderly are fragile in spite of advancement in healthcare and medical attention in Western countries (1). With the global growth of the elderly population, the burden of noncommunicable diseases, including heart disease, cancers, metabolic, neurologic and autoimmune diseases, as well as infectious diseases is projected to dramatically increase, thus imposing critical public health challenges and demanding multidisciplinary efforts to develop more effective preventive and therapeutic strategies. Lying along the causal path between aging and negative health outcomes, a progressive functional decline of the innate and adaptive immunity (immunosenescence) occurs with age, which leads to increased risk for infectious diseases and ineffective response to vaccination, as well as for other non-infectious immune-related chronic degenerative diseases (2).

The unsuccessful immune response to SARS-CoV-2 has been associated with a dysfunctional innate immune response, while neutralizing antibody response by adaptive immunity correlates with beneficial outcomes (3). Aging is accompanied by a decline in the production of naïve T cells and naive B cells, as a consequence of reduced thymic output, bone marrow and lymph nodes function (4). Decreased proliferation and differentiation of activated T and B cells also occur in lymph nodes with aging. Consequently, these age-related changes in the number and function of effector cells impair the immune responses to emerging infection, predisposing older adults to a higher risk of viral and bacterial infections (5).

It has been increasingly shown that this immune dysfunction and associated frailty are, to a relevant extent, accounted for by gender- and age-related changes in the circadian system (6).

## The Circadian System

Biological life is regulated by, and depends on, circadian clocks that allow homeostatic daily rhythms in behavior, physiology, endocrinology and metabolism, with the sleep-wake cycles alternation as the most directly experienced expression of this regulatory system.

The circadian system regulates daily rhythms in sleep-wake, hunger-satiety, and body temperature. Circadian clocks include the central pacemaker situated in the suprachiasmatic nucleus in the brain, and peripheral clocks in various tissues (7). By receiving light inputs via the retina, the suprachiasmatic nucleus is entrained to the 24-h day/night cycle, and in turn synchronizes peripheral clocks. At the cellular level, molecular clock components generate circadian fluctuation in basic cellular functions, e.g., gene expression, protein translation, and intracellular signaling, which are all involved in fundamental processes including cell cycle regulation, nutrient sensing/utilization, metabolism, stress response, redox regulation, detoxification, and cell defense (immunity and inflammation) in a tissue-specific manner (8).

## Gender and Age-Related Changes in Sleep

The cyclic occurrence of periods of non-rapid-eye-movement (NREM) sleep and rapid-eye-movement (REM) sleep characterizes the organization of human sleep. Current evidence confirms the presence of age-related changes in sleep duration and architecture, so that older adults could have a reduced ability to sleep rather than a reduced sleep need (9). Decreases in slow-wave sleep (SWS), slow waves (SW; <4 Hz and >75 μV) and slow-wave activity (SWA; spectral power 0.5–4 Hz) represent one of the main age-related changes in sleep. A factor modifying the impact of aging on sleep is gender. Indeed, women present sharper and steeper SW compared with men. The production of flatter slopes of SW rather than sudden SW slope (200 μV/s) is more frequently observed in older man compared with older women (10). During young adulthood, women reach both puberty and their peak eveningness more precociously compared with men. Sleep is longer in duration in women than in men until age 50–60, that coincides with menopause. Core body temperature and melatonin rhythms are phase advanced, and the intrinsic circadian period is shorter in women compared with men (9). Sleep-endocrine activities also exhibit gender differences and changes in nocturnal hormone secretion occur during aging. The sleep-related secretion of different hormones (particularly neuropeptides and steroids) shows distinct patterns, exerting specific effects on sleep (11): studies in male and female rats found modifications in responses of anterior pituitary to CRH, LHRH, TRH, and GHRH throughout the whole life span (12). Some hormones (GHRH, ghrelin, galanin, NPY) positively regulate sleep, at least in males: GHRH promotes NREM sleep, and stimulates GH. Other peptides (CRH, somatostatin) impair selective NREM sleep: CRH contributes to wakefulness and to the HPA hormones. Notably, in women GHRH induces CRH-like effects. Cortisol secretion is higher in normal young females than in males, and contributes to REM sleep maintenance. Aging as well as some pathological conditions such as depression are accompanied by modifications in the CRH:GHRH ratio with an increase of CRH. In females the menopause is the crucial point for the occurrence of impaired sleep, while in men the sleep quality gets worse steadily: different concurrent decreases of SWS, SWA and GH secretion emerge already during the third decade of life (11). These sleep age-related changes in sleep-endocrine activities, albeit different in both male and females, can impair health and increase the risk for several diseases and infections in elderly populations (13).

## Inflammation, Sickness, and Sleep

Inflammation is a physiological response of the immune system to infection and injury regulated by different immune cells and inflammatory factors. Inflammatory molecules (cytokines, prostaglandins, and complement factors) act locally but also at a distance. Locally, they take action on nearby immune cells to stimulate their recruitment/activation, and on the vascular system to induce vasodilation and vascular permeability. They also communicate over great distances to other organs, establishing a systemic inflammation that can involve brain inflammation, also known as neuroinflammation (14). During infections and injuries, peripheral cytokines can come into contact with the brain via the humoral routes and the neural routes, working in parallel and synergistically to cause neuroinflammation. A strategy to adaptively recover from infections and injuries includes sickness, which induces an inflammatory response associated with physiological and behavioral changes involving disturbed sleep. Evidence point to a reciprocal interactions between sleep and immune function (13), where poor sleep alters immune function and immune activation alters sleep. Inflammatory mechanisms, involving the pro-inflammatory cytokines interleukin (IL) 1α, IL1β, IL6, interferon (IFN)α, IFNγ, tumor necrosis factor (TNF)-α, and TNF-β, are induced by pathogen and are able to modulate sleep organization and main sleep-wake behavior (15). Although IL-1 is commonly associated with pathological states and plays critical roles in host defense, this cytokine is also implicated in the regulation of physiological sleep (15, 16).

## Immunosenescence, Sleep, and Frailty

With aging, the circadian indicators of the sleep-wake organization, i.e., sleep onset, body core temperature, motor activity, and melatonin phase, have an earlier daytime emergence (6); the circadian phase advances in parallel and the rhythms amplitude is reduced while the period is shortened. Evidence from animal studies indicate that the activity of the suprachiasmatic nuclei orchestrating the circadian rhythms is decreased with aging and the clock gene expression is impaired (17, 18); the autonomic nervous system cyclic organization also changes with age (6). In peripheral blood cells, level of Bmal1 expression, a core clock gene and a regulator of innate immunity, was shown to correlate inversely with age in women (19). Animal results further support these age-associated changes in clock gene expression, but also suggest a causal role for these changes in the aging process. For example, knockouts of the clock genes Bmal1 and Period result in an accelerated aging in Drosophila and mice, characterized by increased tissue decline, cognitive dysfunction, and shorter lifespan compared with age-matched wild-type controls (6). On the other hand, the increased susceptibility to respiratory viral diseases in winter may be associated with the seasonal variation of Bmal1 expression, whose low levels in winter correlate with enhanced viral disease (20).

One of the most consistent evidence of age-associated circadian disruption is the age-associated changes in sleep, with its quantity progressively reducing (sleep deprivation) and quality worsening from around 55 yrs. of age to become worst over 65 yrs. also in subjects without major sleep disorders. Major sleep disorders and especially sleep deprivation are associated to immune dysfunction and chronic inflammation and to higher risks of infection and psychiatric, cerebral/cardiovascular, metabolic/hormonal co-morbidity and related mortality irrespective of age, according to surveys and population-based studies (21, 22). Congruently, the disruption of circadian rhythms results in comparable outcomes and in the elderly adds and contribute to the cumulative decline in several (brain, endocrine, immune, muscle) physiological systems during a lifetime.

The circadian system and sleep have emerged as important intertwined regulators of immune defense (23, 24). Trafficking of immune cells and proinflammatory cytokines are under circadian regulation and oscillate in accordance with the rest/activity cycle (5, 25). Animal studies found that global, brain or peripheral knockout of clock genes alters this circadian fluctuation and leads to exacerbated inflammatory response to infection or other pathogenic stimuli, oxidative stress, and age-related phenotypes, thus revealing a direct role for clock genes in suppressing chronic inflammation and ensuring its timely resolution (26, 27).

**Figure 1:**
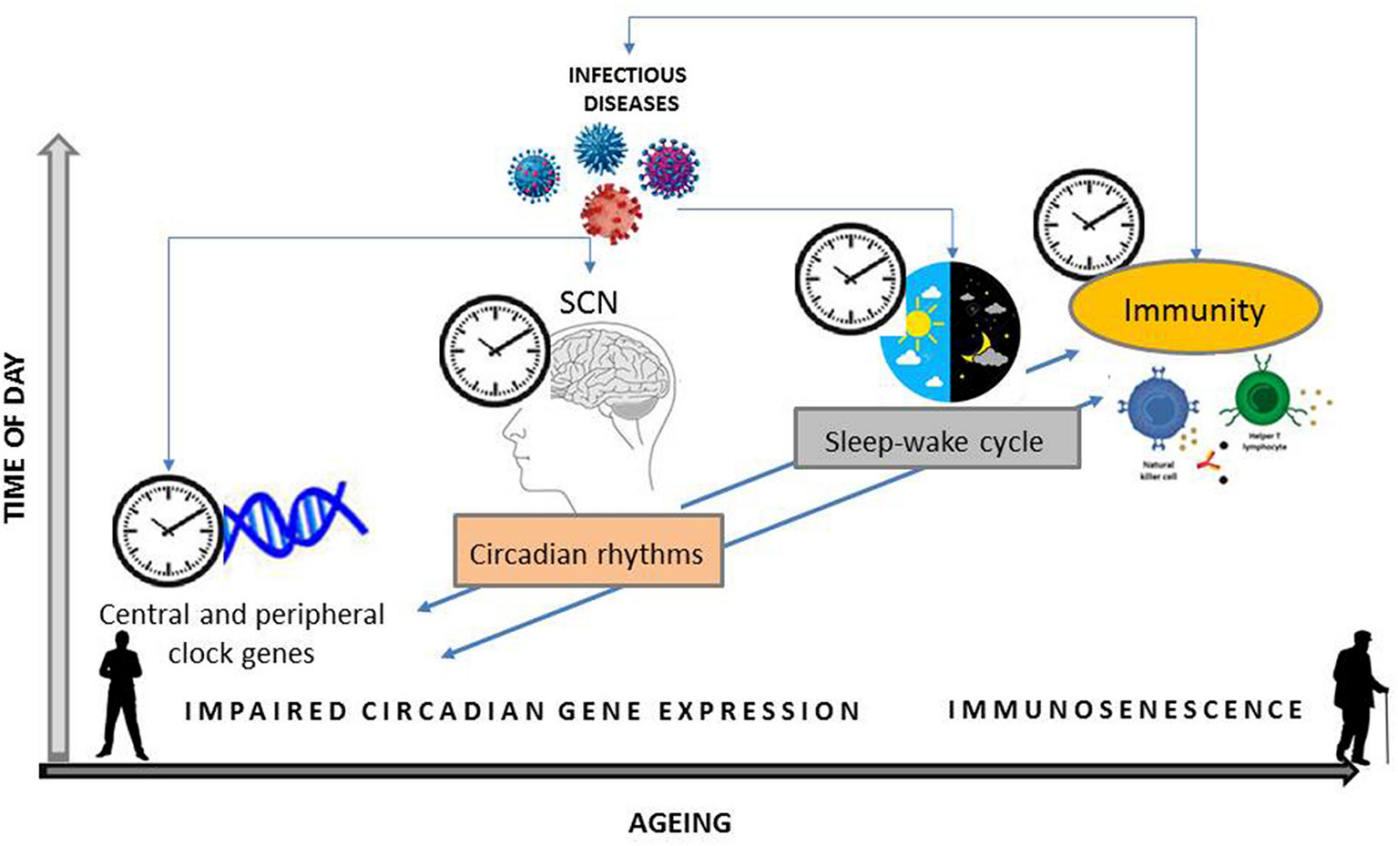
Schematic organization of the bidirectional relationship between central and peripheral clock genes expression and their effects on circadian rhythms, i.e., sleep-wake cycle, and on immunity. Infectious agent can interfere with both the expression of clock genes and the activation of the immune system. Host susceptibility to an invading pathogen modify during 24 h and with aging.

Additionally, adequate sleep quantity and quality support the immune function, reduce infectious risks and improve vaccination responses by regulating immunological memory and humoral immunity (24). On the contrary, sleep deprivation is associated with chronic inflammation, greater susceptibility to infection and worse clinical protection after vaccines (24), as well as with increased risk for inflammatory diseases and total mortality (22). Notably, both sleep-wake cycle and circadian clock components contribute to maintain blood-brain barrier function, important for the neuroimmune system (28). Furthermore, the circadian rhythmicity of gene expression related to immune function and stress response, among others, is disrupted after sleep deprivation, as experimentally demonstrated in humans (29), underscoring the interrelationship between sleep and circadian system and their interface with health. Together, observational and experimental data point to immune dysfunction and chronic, low-grade activation of inflammation in connecting circadian disruption to negative health consequences in aging (Figure 1).

The spatiotemporal regulation of gene expression and associated functions contribute to maintain physiology and behavior.

## Future Directions: Therapeutic Opportunities Thinking About Age-Related Sleep Changes

From accumulating evidence new therapeutic opportunities have been generated for two main reasons: first, by leveraging rhythmic aspects of some diseases in occurrence, including cardiovascular or neurological ones (30) and infection disease also knowing that the timing of virus infection during the day influences infection disease outcome and this effect is found under different types of light–dark cycles (12-h light/12-h darkness compared with 24-h darkness), and in genetic models of circadian disruption (31); second, by optimizing timing of drug administration in order to increase its efficacy and minimize adverse side effects (chronopharmacology) (32). Murine and human studies have suggested that the efficacy of vaccination is under circadian control. Animal studies show that the immune response to vaccination is improved when vaccination is performed at the time of higher circadian expression of TLR9 (Toll-like receptor 9) (33). Likewise, a time dependance of the antibody response to viral challenge in humans was also shown: morning vaccination against hepatitis A and influenza virus increased the antibody response as compared to afternoon vaccination (34). The majority of drugs approved by the FDA have targets showing circadian rhythm in expression and functions (8), and the drug absorption, transportation, conversion and cell uptake also show rhythmic expression. As a consequence, any strategies to improve drug efficacy in the elderly should consider the impact of circadian rhythmicity on treatment efficacy. Chronopharmacology can therefore constitute an additional factor of variability in drug response in the elderly.

## Conclusions

The relationship between aging and circadian disruption is bidirectional, with aging associated with circadian dysfunction, and behavior and genetic disruption of the circadian clock leading to aging-like phenotypes. The result is the establishing of circular, self-sustaining mechanisms of dysregulation with potentially severe impact on health. The bidirectional relationship among sleep/circadian rhythm and immunity has received minor attention to date mainly if we consider the emergence of sleep disorders in the adulthood that persist until the elderly. It remains unclear the extent to which sleep disorders affect the immunosenescence in the later life. During the lifetime, a progressive cumulative decline in physiological homeostasis occurs, thus reducing the quality of life, and promoting isolation and adverse health outcome in the elderly (35). In this subpopulation with combined motility and cognition impairment, multimorbidity, sarcopenia and reduced autonomy (~7–12% in Western countries), the misalignment between the circadian sleep-wake cycle and the daily activities further reduces adaptation and can result in highest incidence of severe adverse medical or traumatic events (36). By decreasing the amplitude in the rhythms of locomotor activity and temperature, circadian rhythmicity could worsen motility, as recently demonstrated in a mouse model of Bmal1 knockout (26).

The link among the disorders of circadian clock, sleep and immunosenescence qualify today as a high-priority healthcare, welfare and social constraints with high estimated direct and indirect costs, that hover higher in the elderly (36) if counterbalancing behaviors or measures are not devised and widely represented in the medical practice. The field of circadian rhythm has recently become a potential therapeutic target for the treatment of diseases as we learned by infectious models, thus improving life expectancy and quality of life of older persons.

The circadian pattern of the immune system contributes to shape the host ability to respond to viral infections and the host–pathogen interaction. Viral infections and other infectious diseases influence the host circadian clock drive. Therefore, in the complex host–pathogen interaction a role of timing can be suggested, offering new therapeutic opportunities based on chrono-modulated antiviral strategies. A more in-depth research in the interrelationship of circadian system and immunity in the elderly, with multidisciplinary competences, from (chrono)biology, (chrono)pharmacology, neuroimmunology, sleep to integrative physiology, is advisable and possibly overdue.

## Author Contributions

SG and WS conceived the manuscript. SG, EG, and PL wrote the manuscript. NB and WS revised the manuscript. All authors contributed to the article and approved the submitted version.

## Conflict of Interest

The authors declare that the research was conducted in the absence of any commercial or financial relationships that could be construed as a potential conflict of interest.
